# Current use of smokeless tobacco among adolescents in the Republic of Congo

**DOI:** 10.1186/1471-2458-10-16

**Published:** 2010-01-14

**Authors:** Emmanuel Rudatsikira, Adamson S Muula, Seter Siziya

**Affiliations:** 1 Division of Epidemiology and Biostatistics, San Diego State University, California, USA; 2 Department of Public Health, Division of Community Health, College of Medicine, University of Malawi, Blantyre, Malawi; 3 Department of Community Medicine, School of Medicine, University of Zambia, Lusaka, Zambia

## Abstract

**Background:**

Tobacco use is a leading cause of global morbidity and mortality. Much of the epidemiologic research on tobacco focuses on smoking, especially cigarette smoking, but little attention on smokeless tobacco (SLT).

**Methods:**

Using data from the Republic of Congo Global Youth Tobacco Survey (GYTS) of 2006, we estimated the prevalence of SLT use among in-school adolescents. We also assessed the association between SLT use and cigarette smoking as well as the traditional factors which are associated with cigarette smoking among adolescents (e.g. age, sex, parental or peer smoking). Unadjusted odds ratios (OR) and adjusted odds ratios (AOR) together with their 95% confidence intervals (CI) were used to measure magnitudes of associations.

**Results:**

Of the 3,034 respondents, 18.0% (18.0% males and 18.1% females) reported having used smokeless tobacco (chewing tobacco, sniff or dip) in the last 30 days. In multivariate analysis, no significant associations were observed between age and sex on one hand and current smokeless tobacco use on the other. Cigarette smokers were more than six times likely to report current use of smokeless tobacco (AOR = 6.65; 95% CI [4.84, 9.14]). Having parents or friends smokers was positively associated with using smokeless tobacco (AOR = 1.98; 95% CI [1.51, 2.59] for parents who smoked cigarettes, AOR = 1.82; 95% CI [1.41, 2.69] for some friends who smoked cigarettes, and AOR = 2.02; 95% CI [1.49, 2.47] for most or all friends who smoked cigarettes). Respondents who reported have seen tobacco advertisement on TV, billboards and in newspapers/magazines were 1.95 times more likely to report current use of smokeless tobacco (AOR = 1.95; 95% CI [1.34, 3.08]). Perception that smoking was harmful to health was negatively associated with current use of smokeless tobacco (AOR = 0.60; 95% CI [0.46, 0.78]).

**Conclusions:**

Prevention programs aimed to reduce teen [cigarette] smoking must also be designed to reduce other forms of tobacco use. The teenagers environment at home, at school and at leisure must also be factored in order to prevent their uptake or maintenance of tobacco use.

## Background

Tobacco is a leading cause of preventable morbidity and mortality globally [1,2]. The most severe adverse health consequences occur many years after initiation of tobacco use. The majority of tobacco users initiate the practice in adolescence or young adulthood. Most of the global reports on tobacco use have been on cigarette use but with limited information available on smokeless tobacco use. While it is likely that the majority of tobacco users smoke cigarettes or other forms of tobacco rather than use smokeless tobacco (SLT), with the absence of data, such claims can be contested. There has however been a surge in SLT products marketing as cigarette and SLT manufacturers as a response to the effect of public health interventions which have threatened the smoking industry [3]. Manufacturers have developed new SLT products to target cigarette smokers promoting dual cigarette and SLT use [3,4]. Smokeless tobacco use may be associated with concomitant tobacco smoking [5], although O'connor et al [6] has reported that the majority of SLT use is not associated with smoking. In addition, smokeless tobacco use has bee associated with periodontal disease, oral cavity and upper gastrointestinal cancers, and hypertension [7-9].

In a small study of 155 Congolese (Congo-Brazzaville) students in 2004, Courtois et al [10] reported that 3.0% and 1.9% of males and females, respectively, were tobacco smokers. Of the 13 to 15 year olds in the 2006 Congolese Global Youth Tobacco Survey (GYTS), 22.0% had ever smoked cigarettes, 23.8% currently use any tobacco products and 11.4% currently smoke cigarettes. While risk factors associated with adolescent cigarette smoking have been extensively studied [11-16], not so much has been reported on the socio-demographic factors that are associated with SLT among African adolescents. We therefore used the Congolese GYTS data of 2006 to estimate the prevalence of SLT use and the socio-demographic factors associated with it. We believe that public health interventions aimed at discouraging tobacco use among adolescents will be much more effective if not only concentrate on cigarette smoking. In addition, knowledge of the factors that may be associated with SLT use may also guide the design and evaluation of intervention as these risk factors will be taken into consideration.

## Methods

The current study was based on secondary analysis of the Republic Congo (Congo-Brazzaville) GYTS data of 2006. A complete description of the survey methods of the GYTS has been reported in the literature [11-17]. In brief however, the survey uses a two stage cluster sample design to select a representative sample of students from schools in the selected country or region. In the first stage of sampling, schools are sampled with the probability of selection being equal to the school's enrollment size; in the second stage, classes within chosen schools are selected using a systematic equal-probability sampling with a random start. All students in the selected classes are eligible to participate in the survey. In addition, participation is voluntary, and self-administered questionnaires are completed anonymously and no personal identifier data are collected. While the school response rate was 100%, the student response rate was 57%, resulting in an overall response rate of 57%.

### Data analysis

Frequencies and proportions (percentages) were calculated for socio-demographic characteristics and also stratified by sex (males versus females). The association between explanatory variables and the outcome (smokeless tobacco use) was assessed via logistic regression analysis. We report unadjusted odds ratios (OR) and adjusted odds ratios (AOR) together with their 95% confidence intervals (CI).

## Results

### Sample description

Table 1 presents selected characteristics of the study population of 3,034 Congolese adolescents (median age 13 years old; Q_1 _=< 12 years and Q_3 _= 15 years). Most of the sample was exposed to tobacco advertisement (91.9%). Almost 1 in 5 respondents (18.0%) reported current use of smokeless tobacco (chewing, snuff or dip).

**Table 1:** Socio-demographic characteristics of the study population

Characteristic	Total 100% (n = 3034)	Males 51.5% (n = 1563)	Females 48.5% (n = 1471)
Age (years)			
≤12	32.0 (1098)	32.2 (559)	31.8 (539)
13	18.5 (463)	18.1 (219)	18.9 (244)
14	18.4 (458)	16.7 (215)	20.2 (243)
15	16.8 (402)	15.7 (208)	18.0 (194)
16-17	14.3 (592)	17.3 (354)	11.0 (238)
Current use of smokeless tobacco			
Yes	18.0 (588)	18.0 (293)	18.1 (295)
No	82.0 (2422)	82.0 (1255)	81.9 (1167)
Current cigarette smoking			
Yes	11.5 (347)	14.1 (220)	8.7 (127)
No	88.5 (2260)	85.9 (1143)	91.3 (1117)
Parental smoking status			
None	71.9 (2108)	70.8 (1077)	73.0 (1031)
One or both parents were smokers	28.1 (902)	29.2 (473)	27.0 (429)
Best friend smoking status			
None	75.6 (2194)	72.8 (1087)	78.5 (1107)
Some	18.1 (602)	20.7 (344)	15.3 (258)
Most or all	6.3 (207)	6.5 (113)	6.2 (94)
Perception that tobacco use is harmful			
No	46.5 (1510)	45.2 (770)	47.7 (740)
Yes	53.6 (1493)	54.8 (777)	52.3 (716)
Seen tobacco advertisement on TV, billboards and in newspapers/magazines in last 30 days			
No	8.1 (185)	7.8 (88)	8.3 (97)
Yes	91.9 (2419)	92.2 (1263)	91.7 (1156)

NB: Percents have been weighted but not the frequencies

### Factors associated with smokeless tobacco use in bivariate analyses

Younger respondents (12 years olds or younger) had higher rates of those who started using smokeless tobacco before the age of 11 years (33.0% ) compared to 19.2% for 13 years olds, 18.0% for 14 years olds, 17.0% among 15 years olds, and 12.8% for 16 years and older. Other factors associated with SLT use in bivariate analyses are shown in Table 2. Participants who reported cigarette smoking were nine times more likely to be current users of smokeless tobacco (OR = 9.00; 7.01, 11.55]). Having parents or friends who smoked cigarettes, and having seen tobacco advertisement (newspapers, billboards and TV) in the last 30 days prior to the survey were positively associated with current use of smokeless tobacco. The perception that tobacco use was harmful to health was negatively associated with current use of smokeless tobacco (OR = 0.61; 95% CI [0.52, 0.74]).

**Table 2:** Current use of smokeless tobacco (chewing, sniff or dip) by age, gender, current cigarette smoking, perception that tobacco use is harmful to health, and having seen tobacco advertisement in last 30 days among Congolese adolescents in 2006

Characteristic	*OR [95% CI]	*AOR [95% CI]
Age (years)		
≤12	1.00	1.00
13	**0.59 [0.44, 0.78]**	0.71 [0.48, 1.12]
14	**0.50 [0.37, 0.67]**	0.61 0.40, 1.03]
15	**0.76 [0.59, 0.94]**	0.75 [0.50, 1.12]
16-17	**0.64 [0.50, 0.82]**	0.68 [0.47, 1.01]
Gender		
Female	1.00	1.00
Male	0.92 [0.77, 1.14]	0.85 [0.62, 1.08]
Current cigarette smoking		
No	1.00	1.00
Yes	**9.00 [7.01, 11.55]**	**6.65 [4.84, 9.14]**
Parental smoking status		
None	1.00	1.00
One or both parents were smokers	**3.52 [2.92, 4.23]**	**1.98 [1.51, 2.59]**
Best friend smoking status		
None	1.00	1.00
Some	**3.52 [2.46, 5.04]**	**1.82 [1.41, 2.69]**
Most or all	**3.67 [3.00, 4.50]**	**2.02 [1.49, 2.47]**
Perception that tobacco use is harmful		
No	1.00	1.00
Yes	**0.61 [0.52, 0.74]**	**0.60 [0.46, 0.78]**
Seen tobacco advertisement on TV, billboards and in newspapers/magazines in last 30 days		
No	1.00	1.00
Yes	**2.34 [1.46, 3.77]**	**1.95 [1.34, 3.08]**

Adjusted models - adjusted for all variables included in Table.

*OR - Un adjusted odds ratio

**AOR - Adjusted odds ratio

### Factors independently associated with smokeless tobacco use

Table 2 also presents adjusted estimates (AOR) from multivariate analysis. Cigarette smoking (AOR = 6.64; 95%CI [4.84, 9.14]), parents (AOR = 1.98; 95%CI [1.51, 2.59]) or friends (AOR = 1.82; 95%CI [1.41, 2.69] for some friends; and AOR = 2.02; 95%CI [1.49, 2.47] for all of the friends) who were smokers, and having seen tobacco advertisement on TV, billboard and in newspapers remained positively associated with current use of smokeless tobacco (AOR = 1.95; 95%CI [1.34, 3.08]). Likewise, the belief that tobacco use is harmful to health remained negatively associated with current use of smokeless tobacco (AOR = 0.60; 95%CI [0.46, 0.78]). In the factor analysis, the final communality estimate for exposure to tobacco advertisement through newspapers, billboards, and TV was 0.94, which is an indication of high inter-correlation between the three variables.

## Discussion

In a secondary study of Congolese school-going adolescents, we found that the prevalence of smokeless tobacco use was 18.0%, with no sex or age differences in the prevalence. Our prevalence of 18.0% among males compares with a prevalence of 16.1% found among high-school males in Karachi, Pakistan [18], and a prevalence of 17.0% among 8^th^-grade males in Missouri, Columbia [19]; but our prevalence is much higher than the 3.6% reported by Kaduri et al in Ilala, Tanzania [20].

In multivariate analysis, current cigarette smoking was positively associated with current use of smokeless tobacco. Similar findings have been reported elsewhere [18,21,22]. Bombard et al [5] have reported that adolescents in the United States were polytobacco users (using more than one tobacco products). The fact that being a current cigarette smoker was associated with smokeless tobacco use is a manifestation of this phenomenon where adolescents who use one form of tobacco are also likely to use other forms. The polytobacco use may also be an example of "clustering" of harmful lifestyles, which we had described in another article [23]. The clustering issue is that adolescents having one harmful lifestyle are more likely to also have other harmful practices in their lives [24].

The fact that other factors associated with cigarette smoking, such as having parents or friends who were smokers, having seen tobacco advertisement on TV, billboard and in newspapers were associated with smokeless tobacco use may not be surprising. Adolescents who are exposed to pro-tobacco media may be sensitized to perceive tobacco use (in all its forms) as not problematic. Similarly, that adolescents who believed that tobacco use is harmful to health were less likely to currently use of smokeless tobacco could be expected that adolescents who perceive tobacco as harmful to health may do so across all forms of tobacco.

Among the strengths of our study are the fact that the sample was large, therefore allowing for precise effect estimated (due to a large power) as well as the fact that the sample was national in scope. However, several limitations exist. The survey participants' reports of SLT use or cigarette smoking were not validated by any biomarkers or interview with friends. To the extent that survey participants misreported, whether intentionally or unintentional, our results may be biased. Secondly, while the reliability of adolescent self-reports on sensitive information such as tobacco use has been validated in the United States [25], the GYTS sample has not been validated in Congo or in sub-Saharan Africa. It is therefore only assumed that the reliability of the GYTS questionnaire is not diminished in African setting. We suggest that future studies attempt to validate the GYTS questionnaire in several African settings. An estimated 43% of the eligible adolescents did not participate in the survey. The main reason was that the participants were not available in school. While this relatively low response has potential to bias results, it is most likely that our estimates are lower than the actual prevalence of SLT use. Out of school children are more likely to use tobacco than in-school adolescents. Finally, the GYTS recruits adolescents who are in school on the day that the particular school is visited. Students who are not in school on a particular day and those adolescents who have dropped out or have never been in school are not included. Our findings may therefore not be representative of the adolescent population in Congo.

## Conclusions

In a study of Congolese in-school adolescents, we found that the prevalence of smokeless tobacco use was 18.0%, with no sex differences in the prevalence. In multivariate analysis, current cigarette smoking, having parents or friends who were smokers, and having seen tobacco advertisement on TV, billboard and in newspapers were positively associated with current use of smokeless tobacco. Prevention programs aimed to reduce teen [cigarette] smoking must also be designed to reduce other forms of tobacco use. The teenager's environment at home, at school and at leisure must also be factored in order to prevent their uptake or maintenance of tobacco use.

## Competing interests

The authors declare that they have no competing interests.

## Authors' contributions

ER designed the data analysis plan, conducted the analysis and participated in the interpretation of the results, ASM participated in the interpretation of the results and led the manuscript drafting effort, and SS conducted some reanalyses, participated in the interpretation of the results, and edited the manuscript. All authors read and approved the final manuscript.

## Pre-publication history

The pre-publication history for this paper can be accessed here:

http://www.biomedcentral.com/1471-2458/10/16/prepub
